# Ex-vivo models of the Retinal Pigment Epithelium (RPE) in long-term culture faithfully recapitulate key structural and physiological features of native RPE

**DOI:** 10.1016/j.tice.2017.06.003

**Published:** 2017-08

**Authors:** Savannah A. Lynn, Gareth Ward, Eloise Keeling, Jenny A. Scott, Angela J. Cree, David A. Johnston, Anton Page, Enrique Cuan-Urquizo, Atul Bhaskar, Martin C. Grossel, David A. Tumbarello, Tracey A. Newman, Andrew J. Lotery, J. Arjuna Ratnayaka

**Affiliations:** a Clinical and Experimental Sciences, Faculty of Medicine, University of Southampton, MP806, Tremona Road, Southampton SO16 6YD, United Kingdom; b Biomedical Imaging Unit, University of Southampton, MP12, Tremona Road, Southampton SO16 6YD, United Kingdom; c Computational Engineering and Design Group, Faculty of Engineering & Environment, Boldrewood Innovation Campus, University of Southampton, Burgess Road, Southampton SO16 7QF, United Kingdom; d School of Chemistry, University of Southampton, Highfield, Southampton SO17 1BJ, United Kingdom; e Biological Sciences, Faculty of Natural & Environmental Sciences, Life Sciences Building 85, University of Southampton, Highfield Campus, Southampton SO17 1BJ, United Kingdom; f Eye Unit, University Hospital Southampton NHS Foundation Trust, Southampton SO16 6YD, United Kingdom

**Keywords:** AMD, age-related Macular Degeneration, BrM, Bruch’s membrane, FAK, focal adhesion kinase, POS, photoreceptor outer segments, PEDF, pigment epithelial derived factor, RPE, retinal pigment epithelium, RP, retinitis pigmentosa, TER, trans-epithelial resistance, VEGF, vascular endothelial growth factor, ZO-1, Zonula Occludens, Retinal Pigment Epithelium (RPE), Bruch’s membrane, In-vitro cultures, Synthetic scaffolds, Electrospinning, Retinopathy

## Abstract

The Retinal Pigment Epithelium (RPE) forms the primary site of pathology in several blinding retinopathies. RPE cultures are being continuously refined so that dynamic disease processes in this important monolayer can be faithfully studied outside the eye over longer periods. The RPE substrate, which mimics the supportive Bruch’s membrane (BrM), plays a key role in determining how well in-vitro cultures recapitulate native RPE cells. Here, we evaluate how two different types of BrM substrates; (1) a commercially-available polyester transwell membrane, and (2) a novel electrospun scaffold developed in our laboratory, could support the generation of realistic RPE tissues in culture. Our findings reveal that both substrates were capable of supporting long-lasting RPE monolayers with structural and functional specialisations of in-situ RPE cells. These cultures were used to study autofluorescence and barrier formation, as well as activities such as outer-segment internalisation/trafficking and directional secretion of key proteins; the impairment of which underlies retinal disease. Hence, both substrates fulfilled important criteria for generating authentic in-vitro cultures and act as powerful tools to study RPE pathophysiology. However, RPE grown on electrospun scaffolds may be better suited to studying complex RPE-BrM interactions such as the formation of drusen-like deposits associated with early retinal disease.

## Highlights

• Damage to the Retinal Pigment Epithelium (RPE) is a key feature of retinopathy.
• We describe 2 substrates which support RPE cultures for long-term studies.
• Substrates were; a polyester transwell membrane and a novel electrospun scaffold.
• Both support RPE cultures with structural and functional features of native RPE.
• Electrospun scaffolds may be better for studying some disease-linked RPE changes.

## Graphical abstract

**Figure:**
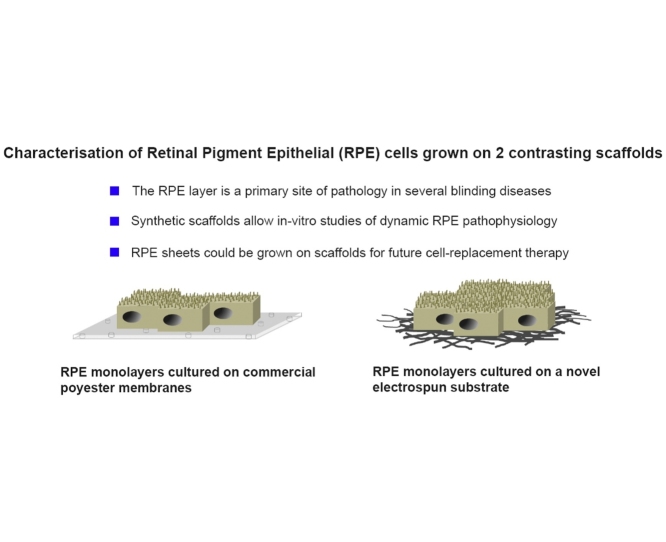

## 1 Introduction

**Fig. 1:**
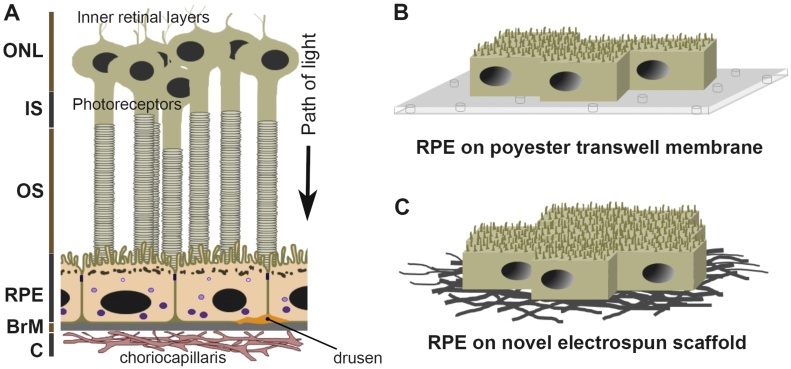
[A] A schematic diagram depicting the arrangement of tissues in the outer retina. The light-sensitive photoreceptors are shown intimately associated with the Retinal Pigment Epithelium (RPE). The RPE monolayer is supported by the Bruch’s membrane (BrM), which lies opposed to the choriocapillaris of the choroid. Protein/lipid deposits referred to as drusen form between the RPE-BrM junction. Their appearance in central retina (macula) is the first clinical indicator of AMD. Outer nuclear layer (ONL), inner segments (IS) and outer segments (OS) of photoreceptors, choroid (C). Endosomal/lysosomal compartments in RPE cells are shown in purple. The path of light is indicated by an arrow. [B–C] A schematic diagram showing long-term RPE cultures on [B] commercially-available polyester transwell membranes and, [C] on a novel electrospun scaffold which was developed in our laboratory.

Degeneration of tissues in the outer retina is involved in ocular pathologies responsible for several blinding conditions. The most prevalent amongst these is Age-related Macular Degeneration (AMD); a complex multifactorial disease with genetic and environmental risks affecting central vision leading to irreversible sight loss. In most cases, the primary site of AMD pathology is considered to occur in the Retinal Pigment Epithelium (RPE) (Khandhadia et al., 2012, Marmor and Wolfensberger, 1998, Lynn et al., 2017), a monolayer of cells underneath the neuroretina, although in some instances disease can also initiate in adjacent tissues (Bird et al., 2014, Spaide, 2009). Examples of rare disorders affecting the RPE include Retinitis Pigmentosa (RP), Sorsby Fundus Dystrophy, Stargardt and Best disease. The outer retina is composed of light-sensitive photoreceptors of the neuroretina which intimately associates with the RPE (Marmor and Wolfensberger, 1998). The RPE in turn sits on a complex pentalaminar tissue of 2–4 μm thickness referred to as Bruch’s membrane (BrM) (Booij et al., 2010), which interface with the subretinal vasculature that provides oxygen/nutrients whilst removing metabolic waste from the outer retina (Fig. 1A). Each tissue therefore carries out a specific set of functions, which becomes impaired with increasing age or onset of disease (Bhutto and Lutty, 2012). Some of the many functions of the RPE monolayer includes (1) internalising and processing shed photoreceptor outer segments (POS) from overlying photoreceptors, and retinoid recycling as part of the visual cycle, (2) fashioning an immunologically privileged ocular environment by creating the blood-retinal barrier, (3) acting as the gatekeeper of metabolic, gas/nutrient exchange in the outer retina, (4) removing free radicals from the highly photo-oxidative retinal environment, and (5) absorbing stray light through pigment granules (Marmor and Wolfensberger, 1998). The pathophysiology of the RPE has therefore garnered considerable attention, and is the focus of this study. Elucidating the manifold mechanisms by which the RPE becomes impaired will provide a better understanding of disease pathways underlying several retinopathies.

Direct analysis of donor eye tissues from patients has provided valuable insights into retinal disease. However, such fixed tissues have limited value for studies into dynamic aspects of RPE pathology, which ideally has to be undertaken in living tissues and/or under in-vivo conditions. The ocular space is also inaccessible to powerful microscopes which could otherwise delve into and ‘capture’ disease-causing events in the RPE in real-time. In order to circumvent these difficulties considerable efforts have been made to recreate ‘authentic’ in-vitro RPE monolayers so that dynamic cellular activities can be studied under experimental conditions. In the past, epithelial cells were cultured on plastic substrates which proved largely unsatisfactory as they failed to reproduce salient structural or functional aspects of native tissues (Widdicombe et al., 2003). It appeared that the substrate on which epithelial cells such as RPE were cultured played a critical role in determining their cellular morphology, behaviour and function. A significant leap in obtaining more authentic RPE monolayers was achieved when cells were cultured on artificial substrates such as porous transwell membranes (Amin et al., 2004, Pfeffer and Philp, 2014). When primary RPE and transformed RPE cell-lines such as ARPE-19 cells (Dunn et al., 1996) were grown on transwell membranes, they not only appeared to exhibit important structural features such as membrane specialisations but also functional abilities of in-situ RPE (Amin et al., 2004, Kannan et al., 2006, Ahmado et al., 2011, Sonoda et al., 2009, Toops et al., 2014). An alternative substrate that could closely recapitulate the BrM may be generated by electrospinning techniques to produce a thin scaffold on which RPE cells can be cultured in-vitro (Treharne et al., 2011). These advanced BrM mimics could also act as a prosthetic support on to which heathy RPE cells could be transplanted in cell-replacement therapy (Treharne et al., 2011, Heller and Martin, 2014, Thomson et al., 2011). Such a prosthetic support by definition must fulfil key criteria of native BrM and be capable of robustly supporting the attachment, spread and differentiation of cells to become a specialised RPE monolayer. Once transplanted back in to the eye, it can therefore effectively replace the diseased counterpart tissue.

Here, using ARPE-19 as well as primary mouse RPE cells, we describe how two different types of substrates could influence the generation of authentic RPE monolayers under in-vitro conditions. We utilized commercially available porous clear-polyester transwell membranes, as well as a novel electrospun BrM scaffold developed in our laboratory (Fig. 1B–C) (Treharne et al., 2012). The electrospun scaffold was specifically developed with inert materials, such that it could act as a potential substrate for future cell-replacement treatments (Treharne et al., 2011, Thomson et al., 2011). Key biophysical characteristics of our first-generation electrospun scaffold have since been modified to create an improved second-generation electrospun substrate which mimics the BrM more faithfully (Ward et al., 2014, Lotery et al., 2016). We asked if differences between the polyester membrane and our electrospun scaffold could play an important role in generating authentic RPE monolayers in culture, and therefore whether some substrates were superior to others. Our findings show that both substrates enable RPE cells to display a considerable repertoire of specialised structural adaptations whilst carrying out salient functions of in-situ RPE. However, our results also suggest that the electrospun scaffold could allow for more realistic in-vitro RPE-BrM assemblies to be generated in-vitro, which may be superior to those on simpler transwell inserts. This first report in which we study RPE structure and function on two very different substrates could help pave the way to establishing more authentic RPE-BrM assemblies in culture. This could enhance our ability to study dynamic disease processes focused around the RPE, thus providing important insights into several blinding retinopathies.

## 2 Materials and methods

### 2.1 Chemical synthesis and electrospinning of artificial RPE scaffold

The copolymer was generated using a combination of methyl methacrylate (MMA; 0.0672 mol, 6.72 g, 7.18 mL), poly(ethylene glycol) methacrylate (PEGM; 0.0125 mol, 4.51 g, 4.08 mL) and azobisiso-butyronitrile (AIBN; Across Organics, Belgium, 0.00058 mol, 0.096 g) as described earlier (Thomson et al., 2011, Treharne et al., 2012, Ward et al., 2014). The copolymer was dissolved in dichloromethane (DCM) solvent and left to stir overnight. The product was then precipitated with diethyl ether and re-dissolved in DCM (Treharne et al., 2012). This solution was drawn into a syringe with a 21 gauge needle (Becton Dickinson, UK) and aligned vertically opposed to a rotating stainless steel collector placed 15 cm from the needle tip. The needle was connected to a high-voltage supply (Glassman FC-series 120 W regulated power supply NJ, USA) set at 16 kV. The syringe pump flowrate was set at 9.5 mL/hour and the resulting electrospun mats were collected and dried under vacuum conditions to remove residual solvent. The thickness of the membrane was reduced from the first-generation electrospun scaffold by decreasing the diameter of individual fibres by altering the voltage supply to 35 kV and the flowrate to 0.05 mL/hour. These alterations also resulted in a tighter weave by reducing the spacing between respective fibres. This was confirmed by diffusion studies and scanning electron microscopy (SEM) measurements as well as scaffold thickness measurements via a ZeScope 3D-optical profilometer (ZeMetrics, AZ, USA) as described before (Ward et al., 2014). Electrospun sheets were cut with a sharp blade into 1 cm^2^ sized pieces which were incubated in fresh tissue culture media and incubated in a 37 °C humidified incubator with 5% CO_2_ for approximately 1 week prior to cell seeding. This acted as a potential washing step and resulted in the scaffold adopting an even/flat surface for subsequent in-vitro cell culture experiments.

### 2.2 Quantification of Young’s modulus

We obtained the Young’s modulus based on the expression valid for thin and slender samples, *E* = tensile stress/tensile strain. Electrospun membranes or human BrM preparations were inserted between the two jaws of a DEBEN MICROTEST tensile tester 300N (Mecmesin Ltd, UK). The initial distance between the two jaws was 1 cm, and the maximum stroke was set at 1 cm with a loadcell of 300N. Samples were mounted horizontally and the device set on displacement control moving the free jaw at a strain rate of 0.1 mm/min. Stress was calculated using the force transducer reading and dimensions of the film shaped samples, whereas strain from the displacement of the jaws of the tensile test stage was measured by a capacitive displacement transducer and the initial length of the sample. The device was regulated electronically with software controlling the motor as well as parameters such as motor speed and sampling time. Data for the force and elongation were obtained and MATLAB software (MathWorks, Inc. USA) used to generate stress-strain curves and also to condition the data for reducing noise by using a procedure that involved averaging over a moving time window.

### 2.3 Cell culture

#### 2.3.1 Primary mouse RPE

Postnatal day 10–12 male and female C57BL/6 mice were obtained from the Biomedical Research Facility (University of Southampton, UK). Animals were housed under temperature and humidity controlled conditions with a 12 h dark/12 h light cycle, and were allowed access to water and standard laboratory chow ad libitum. Animal husbandry and procedures were performed within the guidelines of the University of Southampton Research Ethics Committee and complied with the ARVO (Association for Research in Vision and Ophthalmology) statement for the use of animals in ophthalmic and vision research. Mice were culled under schedule 1 procedures (cervical dislocation confirmed by severing the carotid artery) as outlined in the UK Home Office code of practise on the humane killing of animals. Whole eyes were enucleated immediately post euthanasia and placed in a solution of HBSS without Ca^2+^, Mg^2+^ or phenol red (Life Technologies, UK). The anterior pole was removed by incision. The retina was removed by severing the optic nerve and whole sheets of pigmented RPE removed using a Dumont 5SF tweezer under a dissecting stereomicroscope. In order to maintain high levels of cell viability the procedure was carried out rapidly with only 2 eyes enucleated and prepared as described by Fernandez-Godino and colleagues (Fernandez-Godino et al., 2016). Cells from 2 mouse eyes were pooled in a 15 mL tube and centrifuged at 285*g* for 4 mins at room temperature. The pellet was resuspended in 1 mL 0.05% trypsin and 0.02% EDTA and incubated for 1 min in an incubator set at 37 °C. Cells were washed in fresh culture media consisting of MEM-α (Sigma Aldrich, UK) containing N1 supplement, 1:100 (mL/mL) glutamine-penicillin-streptomycin (Sigma Aldrich, UK), 1:100 (mL/mL) non-essential amino acid solution (Sigma Aldrich, UK), 5–15% heat-inactivated foetal bovine serum (FBS) (Sigma Aldrich, UK) and THT supplement (Sonoda et al., 2009). RPE cells isolated in this manner from mice older than 10 days were reported to be suitable for preparing RPE cultures (Fernandez-Godino et al., 2016). Primary cells from 2 mouse eyes were initially cultured in 24-well sized plates at 80–90% confluency in medium containing 15% FBS, prior to seeding at 1 × 10^5^ cells/well in 6-well sized transwell inserts, or alternatively on 1 cm^2^ sized electrospun scaffolds, at which point FBS levels were reduced to 5%. Cell proliferation was minimised in order to retain intracellular autofluorescent material and minimise genotypic or phenotypic drift. Twice a week, the media was changed as follows. For transwell inserts a complete apical media change and 20% basal media change twice a week. An 80% media change twice a week in dishes with cell-seeded electrospun scaffolds.

#### 2.3.2 ARPE-19

The spontaneously transformed human RPE cell line, ARPE-19 (Dunn et al., 1996) was obtained from the American Tissue Culture Collection (ATCC, USA). ARPE-19 cells were routinely cultured in Dulbecco’s modified Eagle’s Medium (DMEM) with 4.5 g/L l-d glucose, l-glutamine and pyruvate (Life Technologies, UK), supplemented with 1% heat-inactivated FBS (Sigma Aldrich, UK) and 100 units/mL penicillin, 100 μg/mL streptomycin (Ahmado et al., 2011) (Sigma-Aldrich, UK) in a 37 °C humidified incubator with 5% CO_2_. Post confluent ARPE-19 cultures were maintained in T25 flasks for up to 4 months prior to seeding on 0.4 μm pore-sized polyester transwell inserts (Transwell, Corning Costar, UK) pre-coated with 50 μg/mL fibronectin (Sigma-Aldrich, UK). ARPE-19 cells were seeded at a density of either 5 × 10^4^ cells/well for 24 mm diameter inserts, or 1.25 × 10^4^ cells/well for 12 mm diameter inserts, and maintained in 2 mL apical, 3 mL basal media, or 0.5 mL apical, 1.5 mL basal media respectively. Cells were cultured for a minimum of two months prior to use in experiments with a complete apical media change and 20% basal media change twice weekly. ARPE-19 cells used in experiments were between passages 23–27.

### 2.4 Preparation of Human Bruch’s membranes

10 healthy human eyes between ages 71–99 years and within a 24-48 h post-mortem period were obtained from the Bristol Eye Bank (United Kingdom). Following preliminary fundus examination to ensure that eyes were free of disease, BrM-choroid samples were prepared as described previously (Hussain et al., 2011). The donor tissues were managed in compliance with the Declaration of Helsinki for research involving human tissues.

### 2.5 Confocal-immunofluorescence

Mature RPE cultures were fixed in 4% formaldehyde for 30 min at 4 °C, permeabilised and blocked in phosphate-buffered saline (PBS) containing 5% normal goat serum and 0.1% Triton X-100 for 1 h at room temperature and incubated with primary antibody diluted in the same solution at 4 °C overnight. Cultures were probed with the following primary antibodies; rabbit anti-ZO-1 (RRID: AB_2533456, 1:100), mouse anti-RPE65 (RRID: 1566691, 1:100), mouse anti-Na^+^/K^+^ ATPase α-1 (RRID: 306023, 1:100), rabbit anti-RAB5 (RRID: AB470264, 1:200), rabbit anti-RAB7 (RRID: AB_2629474, 1:200), rabbit anti-LAMP1 (RRID: AB_775981, 1:1000), rabbit anti-LAMP2 (RRID: AB_775981, 1:1000), rabbit anti-LC3B (RRID: AB_881433, 1:200), rabbit anti-FAK (AHO0502, 1:100), mouse anti-αVβ5 (AB_24694, 1:100) and rabbit anti-MerTK (AB_95925, 1:100). Following primary antibody incubation, cells were incubated for 1 h at room temperature with Alexa Fluor-conjugated secondary antibody (RRID: AB_2534114, AB_2534064, AB_2534115, AB_2534085, AB_2534116, AB_141974) at a dilution of 1:100 in PBS with 0.05% Triton X-100 and Cytopainter Phalloidin-iFluor 647 (Abcam, AB_176759, 1:1000). In all cases, 1 μg/mL of 4′,6′-diamino-2-phenylindole (DAPI) was used to visualise cell nuclei. Images were acquired using a Leica SP5 or Leica SP8 (Leica Microsystems, UK) confocal laser scanning microscope.

### 2.6 Photoreceptor feeding assay

Retinas were isolated from porcine eyes and pooled in KCl buffer (0.3 M KCl, 10 mM HEPES, 0.5 mM CaCl_2_, 1 mM MgCl_2_) in 48% sucrose solution and homogenised gently for 2 min. The solution was then centrifuged at 5000 × *g* for 5 min before the supernatant was passed through a sterile gauze into fresh centrifuge tubes and diluted with KCl buffer without sucrose. This preparation was subsequently centrifuged at 4000 × *g* for 7 mins, following which the pellet was washed three times in PBS through further centrifugation at 4000 × *g* for 7 mins. POS were resuspended in 20 mM phosphate buffer (pH 7.2) with 10% sucrose and 5 μM taurine, prior to covalently attaching the FITC conjugate (ThermoFisher, UK). Following addition of the FITC stock (10 mg/mL), the solution was left rotating on a plate for 1 h in the dark. The POS-FITC solution was then centrifuged at 3000 × *g* for 5 mins at 20 °C, resuspended in DMEM with 2.5% sucrose, aliquoted and stored at −80 °C. To quantify POS yields, a BCA protein assay (Pierce™, ThermoFisher, UK) was carried out in which POS were measured against protein standards between 20 and 2000 μg/mL using absorption at 562 nm (spectrophotometer, Infinite F200 Pro, Tecan, Switzerland). RPE monolayers were fed with POS using a pulse-chase method. Briefly, RPE-BrM assemblies were chilled to 17 °C for 30 min (Hall and Abrams, 1987) before being fed with 4 μg/cm^2^ of POS-FITC (Krohne et al., 2010). Cells were left at 17 °C for a further 30 min to allow for maximal binding with minimal internalisation. Following this, the medium was completely aspirated to remove unbound POS and replaced with fresh pre-warmed medium and the cells returned to a humidified incubator at 37 °C with 5% CO_2_.

### 2.7 Electron microscopy

#### 2.7.1 Transmission electron microscopy

Transmission electron microscopy (TEM) was used to evaluate the ultrastructure of RPE monolayers. Briefly, 3–10 month old RPE cultures grown on polyester transwell membranes or on electrospun scaffolds were fixed with primary fixative comprising 3% glutaraldehyde, 4% formaldehyde in 0.1 M PIPES buffer (pH 7.2) for a minimum of 1 h. Specimens were then rinsed in 0.1 M PIPES buffer, post-fixed in 1% buffered osmium tetroxide (1 h), rinsed in buffer, block stained in 2% aqueous uranyl acetate (20 min), dehydrated in an ethanol series and embedded in Spurr resin (Agar Scientific, Stanstead, UK). Silver/gold sections were cut on a Reichert Ultracut E ultramicrotome (Leica Microsystems, UK), stained with Reynolds lead stain and viewed on a Hitachi H7000 (Hitachi High Technology, Japan) fitted with a SIS Megaview III plate camera (EMSIS, Germany).

#### 2.7.2 Scanning electron microscopy

Human Bruch’s membrane preparations and RPE-BrM assemblies on electrospun scaffolds were processed for scanning electron microscopy as described previously (Treharne et al., 2012, Ward et al., 2014). Briefly, samples were fixed with 3% glutaraldehyde and 4% formaldehyde (pH 7.2) in 0.1 M PIPES buffer for 1 h at room temperature and freeze-dried for 5 h before mounting on stubs and sputter coating in gold-palladium. Images were collected using a Quanta 200 (FEI, Eindhoven, Netherlands) scanning electron microscope.

### 2.8 Measurement of *trans*-epithelial electrical resistance

Trans-epithelial electrical resistance (TER) of ARPE-19 and primary mouse RPE cells grown on 12-well transwell inserts (12 mm diameter, 0.4 μm pore size, Corning, UK) were measured as an indicator of barrier integrity/polarisation using an EVOM^2^ epithelial voltohmmeter and 4 mm STX2 chopstick electrode according to the manufacturer’s instructions (World Precision Instruments Inc., FL, USA). Briefly, the electrode was sterilised in 70% ethanol, rinsed in ddH_2_0 and equilibrated in pre-warmed culture medium before being simultaneously introduced into both chambers. TER measurements were taken from at least three cultures over a period of 12 weeks. Reference values from inserts coated with 50 μg/mL fibronectin were subtracted from experimental values to give net TER measurements which were subsequently corrected for the cell growth area of the transwell insert. All measurements were performed within 6 min at room temperature after removal from the incubator. A full media change in transwell chambers was carried out post experimentation to minimise the likelihood of contamination.

### 2.9 Enzyme linked immunosorbent assays

Novex^®^ human vascular endothelial growth factor (VEGF) (Life Technologies, UK) and human pigment epithelial derived growth factor (PEDF) (BioVendor, Germany) enzyme-linked immunosorbent assays (ELISA) were used to quantify levels of secreted VEGF-A 165b and PEDF from ARPE-19 cultures as follows. Three days after a compete media change, conditioned media were collected from apical and basal compartments respectively of 7 week old ARPE-19 cultures grown on 24 mm transwell inserts. ELISA quantification was carried out in triplicate on n = 3 samples following the manufacturer’s instructions. A microtiter plate reader (FLUOstar Optima; BMG Labtech, UK) was used to measure optical densities at 405 nm within 1 h after addition of stop solution and incorporating the wavelength correction at 570 nm. The VEGF and PEDF concentrations were then determined from standard curves and were normalised for different apical and basal transwell volumes. A similar approach was used to quantify VEGF from primary mouse RPE grown on electrospun scaffolds for approximately 9 months. Three days after a compete media change, conditioned media were collected and diluted 1:65 and an ELISA kit specific to murine VEGF (ab100751) was used following the manufacturers’ instructions.

### 2.10 Autofluorescence measurements

Autofluorescence spectra of 5 month old primary mouse RPE cultures as well as 1 and 3 month old ARPE-19 cultures on transwell inserts were obtained via Leica SP5 laser scanning confocal microscope (Leica Microsystems, UK). Lambda scans were acquired across the visible light spectrum at approximately 10–30 nm intervals by excitation with 458 nm, 476 nm, 488 nm, 496 nm, 514 nm, 561 nm, 594 nm and 633 nm lasers. Background spectral scans from fibronectin coated polyester transwell membranes alone were subtracted from acquired values. Spectral values were exported from the Leica LASAF software into an Excel spreadsheet and used to generate graphs.

### 2.11 Statistics

Statistical analyses were performed using GraphPad Prism Software (GraphPad, CA, USA). The statistical significances of differences was determined by the unpaired student’s *t*-test. Data are expressed as means ± standard error of the mean (SEM) with statistical significance denoted as * for p ≤ 0.05 and ** for p ≤ 0.01.

## 3 Results

### 3.1 Development of the electrospun scaffold

We had earlier described how a combination of methyl methacrylate and poly(ethylene glycol) was used to generate an electrospun scaffold. We cultured ARPE-19 cells on this first generation electrospun scaffold for approximately 2 weeks which required the presence of *N,N*’-disuccinimidyl carbonate and RGD (l-arginine, glycine, l-aspartic acid) peptides to facilitate cell attachment. Measurement of individual fibre dimeters and overall thickness of the scaffold by an electro-profilometer and SEM yielded values of 1.9 μm and 50 μm, respectively (Treharne et al., 2011, Thomson et al., 2011, Treharne et al., 2012). However, the thickness of this scaffold was a potential criticism since it was considerably thicker than 2–4.7 μm reported for human BrM (Booij et al., 2010). We therefore addressed this issue as a matter of priority before undertaking any further cell culture experiments. By maintaining a constant distance of 15 cm between the syringe and collector during synthesis, but reducing the flowrate of the copolymer from 9.5 mL/Hr to 0.05 mL/Hr and increasing the voltage applied across the Taylor cone during electrospinning from 16 kV to 35 kV, we successfully reduced the diameter of individual fibres to 0.1–0.8 μm and the overall thickness of the scaffold to 12.3 μm (supplementary Table S1–S2). This improved membrane was referred to as the second generation scaffold (Ward et al., 2014). Next, we studied the effects of reduced fibre dimeter and reduction of scaffold thickness by measuring the ability of FITC conjugated dextran to traverse through the membrane. Our findings show that porosity of the second generation scaffold was similar to human BrM (supplementary Fig. S1). We then assessed the Young’s modulus of the two electrospun scaffolds and compared them to human BrM preparations. This yielded values of 13.6 MPa ± 3.65 for first generation scaffold, 26.0 MPa ± 3.14 for the second generation scaffold and 33.5 MPa ± 4.78 for human BrM preparations. In a further change, the improved scaffold was also made without the RGD peptide modification that was present in the first generation membrane. Hence we sought to determine whether this second generation scaffold could support the attachment and growth of cells, and particular of primary RPE cells. To achieve these objectives, primary mouse RPE cells were seeded on second generation scaffolds with/without RGD modifications. Visualisation of cell spread by confocal immunofluorescence studies revealed that the absence of these modifications made no difference to the ability of primary mouse RPE to effectively attach and proliferate on our improved scaffolds (Ward et al., 2014) (data not shown). Hence, incorporation of the RGD peptides was excluded from second generation electrospun scaffolds, which were used in all subsequent experiments.

### 3.2 RPE cells on artificial scaffolds recapitulate key structural adaptations of native RPE

**Fig. 2:**
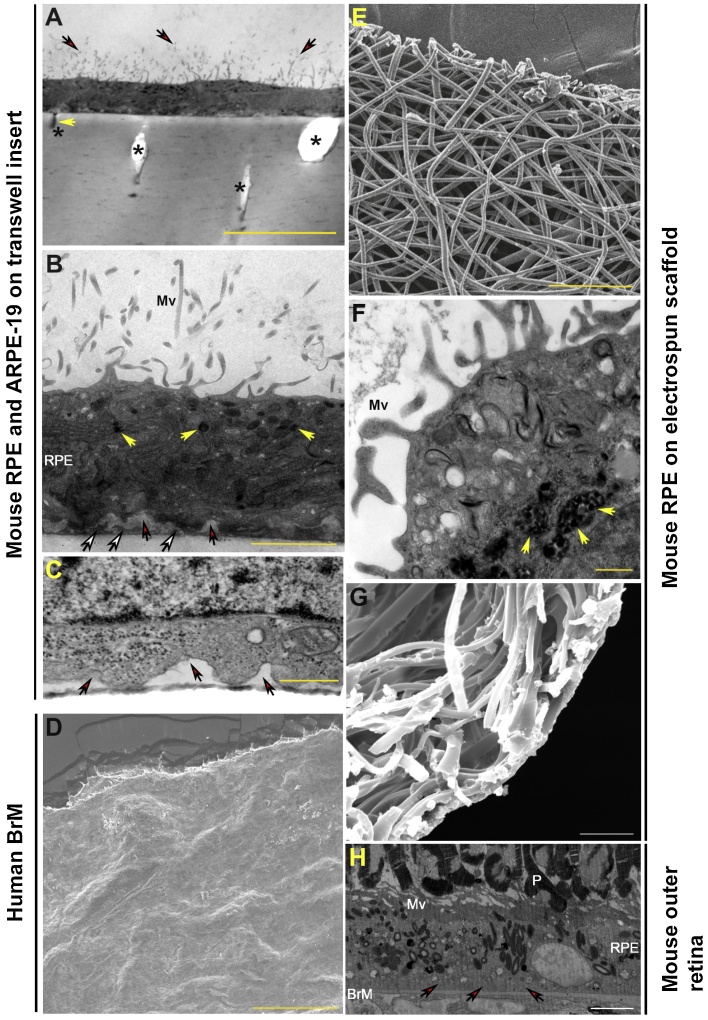
RPE cells on a polyester transwell membrane and on an electrospun scaffold display structural specialisations of native RPE. [A–C] Representative electron micrographs show cross-sections of primary mouse RPE and ARPE-19 cells on transwell membranes. [A–B] Presence of apical microvilli on ARPE-19 (Mv: red arrows in A) and [B–C] micro-infolds (red arrows) on basolateral membranes of [B] ARPE-19 and [C] primary mouse RPE. [A] Cells appear to form projections (yellow arrow) into accessible pores (indicated by *) in the polyester membrane. [B] Electron-dense pigment granules are visible distributed along the apical region (yellow arrows), whilst mitochondria appear predominantly in the mid-basolateral region of cells. Focal sub-RPE deposits are visible under basolateral infolds and on the polyester transwell membrane (white arrows). Scale bars correspond to 10 μm, 2 μm and 500 nm in A, B and C, respectively. [D] Scanning electron micrograph showing top-down view of a human BrM preparation. Scale bar represents 200 μm. [E] Magnified scanning electron micrograph of our second-generation electrospun scaffold showing arrangement of the supportive fibre meshwork. Scale bar corresponds to 100 μm. [F] Electron micrograph showing primary mouse RPE displaying apical microvilli (Mv), and ingesting fed photoreceptor outer segments (yellow arrows). Scale bar corresponds to 200 nm. [G] Representative scanning electron micrograph of 10 month-old electrospun scaffold observed from below; showing deposition of a confluent acellular extracellular matrix over the supportive fibre meshwork. Gaps within fibres appear to contain focal deposits of extracellular material. Scale bar corresponds to 20 μm. Notice the acellular extracellular material laid down by primary mouse RPE on electrospun fibres [G] and the acellular human BrM [D]. [H] Low-magnification transmission electron micrograph showing arrangement of the native mouse RPE monolayer juxtaposed between overlying photoreceptor outer segments (P) and the underlying BrM. Apical RPE microvilli (Mv) and basal micro-infolds (red arrows) are observed along with the intracellular distribution of electron-dense pigment granules. Scale bar corresponds to 5 μm. (For interpretation of the references to colour in this figure legend, the reader is referred to the web version of this article.)

Primary mouse RPE and ARPE-19 cells cultured on polyester transwell membranes (Fig. 2A–C), and primary mouse RPE cells on electrospun scaffolds (Fig. 2E–G), rapidly spread to form a monolayer on respective substrates. These RPE cultures proved long-lasting and robust. For instance, ARPE-19 cells on polyester transwell membranes could be maintained for up to 6 months in culture, whilst primary mouse RPE could be cultured for even longer (up to 12 months) on either polyester transwell membranes or on electrospun scaffolds. Such mature RPE cultures displayed apical microvilli of ∼5 μm in length, convoluted basal micro-infolds as well as electron-dense pigmentation (Fig. 2A–C and F). Polyester transwell membranes enabled RPE cells to establish a compact monolayer with these features more rapidly, in approximately 8 weeks, compared to RPE cells on electrospun scaffolds which took almost twice as long (data not shown). The intracellular distribution of organelles in established RPE monolayers were observed to follow a broad pattern of stratification. For instance, pigment granules were distributed apically, whilst mitochondria were found in mid to lower cellular regions (Fig. 2B, C, F), similar to in-situ RPE (Fig. 2H). However cultures differed with regards to their interaction with respective substrates. The basolateral RPE surface was tightly bound to the underlying polyester transwell membrane and appeared to form cellular projections into accessible pores (Fig. 2A). Focal sub-RPE deposits could also be observed under basolateral micro-infolds (Fig. 2B). By contrast, primary mouse RPE cells cultured on the electrospun scaffold behaved somewhat differently. For instance, the RPE monolayer appeared to interact in a more complex manner with the tightly-woven fibre meshwork underneath. Hence, SEM images of such RPE-BrM assemblies after 6 months revealed what appeared to be a sheet of extracellular material spread over the underlying fibres (Fig. 2G). This extracellular sheath appeared to have been laid down de-novo, and had the appearance of an acellular human BrM (Fig. 2D). SEM studies also revealed apparent extracellular deposits beneath the extracellular sheath; amongst the fibre meshwork (Fig. 2G).

### 3.3 Ex-vivo RPE cultures express key functional elements critical to their role

**Fig. 3:**
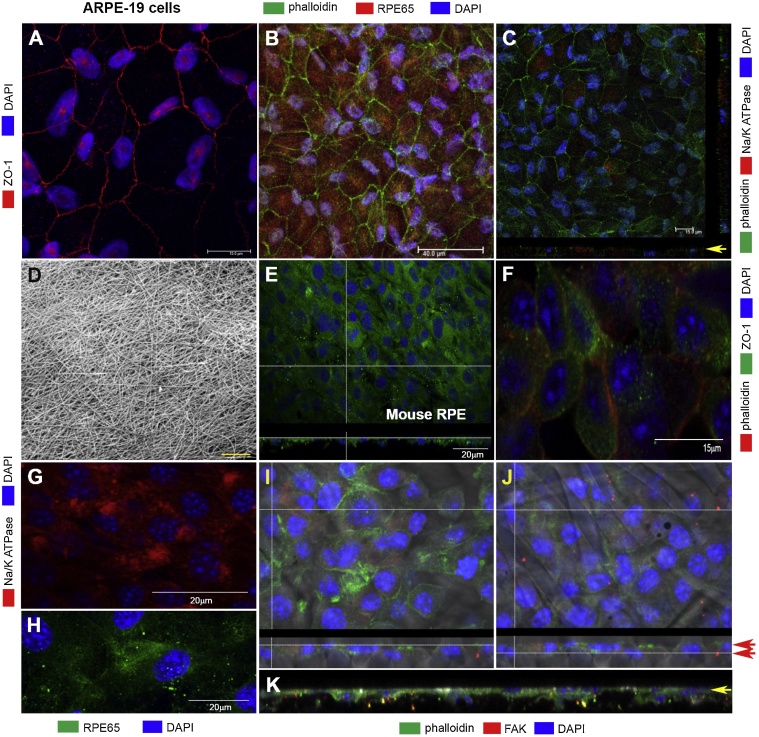
ARPE-19 and primary mouse RPE cells express important functional elements when cultured on different substrates. [A] Representative image from 2 to 4 month old ARPE-19 culture on transwell polyester membranes show junctional proteins ZO-1 (red) as well as [B] the cell-specific RPE65 protein (red). Nuclei and phalloidin appear in blue and green, respectively. [C] Furthermore, these monolayers express the apically polarised NA^+^/K^+^ATPase transporter (red) visible in top-down and orthogonal perspectives (yellow arrow indicating apical RPE membrane). Cytoskeletal F-actin is labelled with phalloidin (green) and nuclei with DAPI (blue). Scale bars in A and C corresponds to 15 μm, and 40 μm in B. [D] Scanning electron micrograph showing the electrospun fibre meshwork of our second-generation scaffold. Scale bar corresponds to 200 μm. [E] Primary mouse RPE initially attach and spread on the scaffold surface to form a confluent monolayer by 4 months. On-face and orthogonal views through a single optical plane showing RPE layer stained with cytoskeletal F-actin (green) and nuclei with DAPI (blue). Scale bar corresponds to 20 μm. [F] Primary mouse RPE on such scaffolds adopts a characteristic hexagonal morphology with ZO-1 junctions (green) and [G] express the NA^+^/K^+^ATPase transporter (red). Phalloidin in [F] appear red whist nuclei DAPI in [F-G] are in blue. Scale bars in F and G correspond to 15 μm and 20 μm, respectively. [H] Mouse RPE also expresses the RPE65 protein (green). Nuclei labelled with DAPI (blue) whilst scale bar corresponds to 20 μm. [I-J] Top-down and corresponding orthogonal view showing confocal Z-stack through a 10 month-old RPE-BrM assembly. Cytoskeletal F-actin (green) in apical-mid RPE cellular regions [I] gives away to reveal points of FAK attachments (red) in the basolateral membrane with underlying fibres [J]. FAK labelling can be observed to overly the arrangement of individual fibres underneath. The two Z-planes are indicated by relative positions of red arrows in orthogonal sections. Fibres are visible in brightfield, whilst RPE cell nuclei are labelled with DAPI (blue). [K] A magnified, high-resolution orthogonal section of a representative 10 month-old RPE-BrM assembly showing basolateral FAK distribution (red) within the RPE monolayer (indicated by yellow arrow). Phalloidin and cell nuclei appear in green and blue, respectively. (For interpretation of the references to colour in this figure legend, the reader is referred to the web version of this article.)

We next assessed whether mature ARPE-19 monolayers on polyester transwell membranes possessed key functional elements necessary for their function. Confluent RPE cultures were found to express ZO-1 junctional complexes (Fig. 3A). RPE cells also expressed cytoplasmic RPE65 (Fig. 3B) as well as the apically localised Na^+^/K^+^ATPase transporter (Fig. 3C) characteristic of this epithelium (Reichhart and Strauss, 2014). We next studied how primary mouse RPE cells attached and spread across the electrospun scaffold (Fig. 3D). During the first 4 months in culture we noticed occasional gaps in the RPE monolayer, presumably where there were no underlying fibres to support cell attachment (data not shown). However, when the same regions were imaged in cultures older than 4 months, these gaps had largely been filled to create a confluent cell-monolayer (Fig. 3E). Primary RPE on electrospun scaffolds also expressed ZO-1 (Fig. 3F), the Na^+^/K^+^ATPase transporter (Fig. 3G) as well as RPE65 (Fig. 3H). We next assessed how primary RPE cells interacted with the underlying fibre meshwork which supported them. Cells attached to fibres expressed via focal adhesion kinases (FAK) on their basolateral surfaces (Fig. 3I–J). The relationship between RPE cells and the electrospun scaffold is evident in cross-section; showing basolateral FAK that forms attachment with the underlying fibre meshwork (Fig. 3K).

### 3.4 RPE monolayers bind photoreceptor outer segments via receptor-mediated interactions followed by internalisation and trafficking via the endocytic-lysosomal pathway

**Fig. 4:**
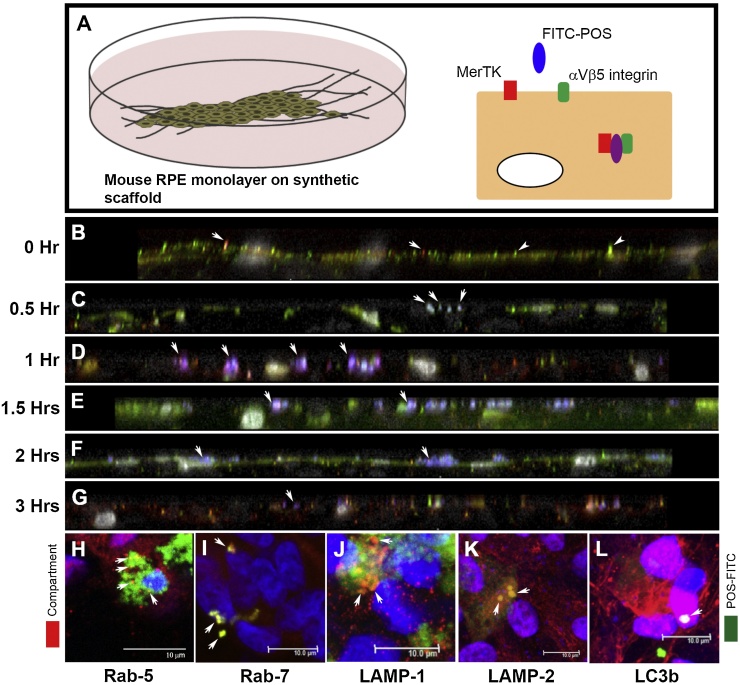
RPE cells on BrM substitutes appear to internalise outer segments through receptor-mediated mechanisms and subsequently via the endocytic-lysosomal pathway. [A] Schematic diagram depicting arrangement of primary mouse RPE cells on electrospun scaffold and outer segment feeding assay. POS: photoreceptor outer segments tagged to FITC. [B] Representative image showing orthogonal profile of a 10 month old RPE culture on our scaffold with apically expressed αVβ5 integrin (green) and MerTK (red) receptors (white arrows). Nuclei appear white. RPE cells follow undulating surface of the electrospun scaffold. [C] POS tagged with FITC (pale blue) can be observed bound to RPE approximately 30 min after challenge. [D-F] Between 1-2 h after initial feed, internalised POS is observed merged with MerTK (red) and αVβ5 (green) to appear as discrete purple coloured puncta (white arrows). [G] By 3 h, ingested POS had largely disappeared, although some purple coloured complexes are occasionally observed. [H-L] Following POS challenge, its intracellular itinerary was tracked through different compartments using 10 week old ARPE-19 cultures on polyester transwell membranes. [H] Representative confocal images showing Rab-5 early endosomes (red) and [I] Rab-7 late endosomes (red) with POS-FITC (green) to appear co-localised as yellow vesicles (white arrows). [J-K] Lysosomal markers LAMP-1 and 2 (red) carrying POS-FITC (green) appear as yellow vesicles (white arrows). [L] 48 h after initial POS feed, POS-FITC (green) positive autophagy marker LC3b (red) appears as yellow vesicles (white arrow). [H-L] RPE nuclei are labelled with DAPI (blue). Scale bars correspond to 10 μm. (For interpretation of the references to colour in this figure legend, the reader is referred to the web version of this article.)

A key function of RPE cells is the daily engulfment and processing of shed POS from overlying photoreceptors (Khandhadia et al., 2012, Marmor and Wolfensberger, 1998, Bhutto and Lutty, 2012). We sought to determine whether mature cultures of primary mouse RPE cells on electrospun scaffolds were capable of carrying out this vital task. Challenge of RPE-BrM assemblies with POS in a feeding assay (Fig. 4A) resulted in POS-FITC binding via apically-localised tyrosine kinase MerTK and integrin αVβ5 receptors (Fig. 4B). This was followed by rapid receptor-mediated internalisation of POS soon after (Fig. 4C–G). Mature cultures of ARPE-19 cells on polyester transwell membranes were also capable of carrying out this important function (Figs. S2–S3). Once POS was internalised by ARPE-19 cultured on polyester membranes we sought to determine how cargos were intracellularly trafficked via distinct compartments in the phagocytic pathway. We observed that POS-FITC was initially trafficked through early endocytic Rab-5 vesicles (Fig. 4H), followed by late Rab-7endosomes (Fig. 4I) and in LAMP-1 (Fig. 4J) and LAMP-2 (Fig. 4K) positive lysosomes. For instance, cargos could be observed in Rab-5 positive endosomes as early as two hours after commencing the pulse-chase experiment. By 24 h, all cargos had passed through endosomes as well as lysosomes. 48 h after feeding, POS-FITC were observed in LC3b positive vesicles (Fig. 4L), indicating an accumulation in RPE autophagosomes. We also measured the average diameter of each membrane-bound vesicle type in 3 separate confocal z-stack images/compartment. This yielded values of 318 nm ± 69.2 for Rab-5 vesicles, 421 nm ± 19.4 for Rab-7 vesicles, 677 nm ± 32.1 for LAMP-1 vesicles, 712 nm ± 59.0 for LAMP-2 vesicles and 990 nm ± 23.9 for LC3b autophagic bodies.

### 3.5 Mature RPE monolayers maintain a stable *trans*-epithelial barrier and exhibit directional secretion of key proteins in culture

**Fig. 5:**
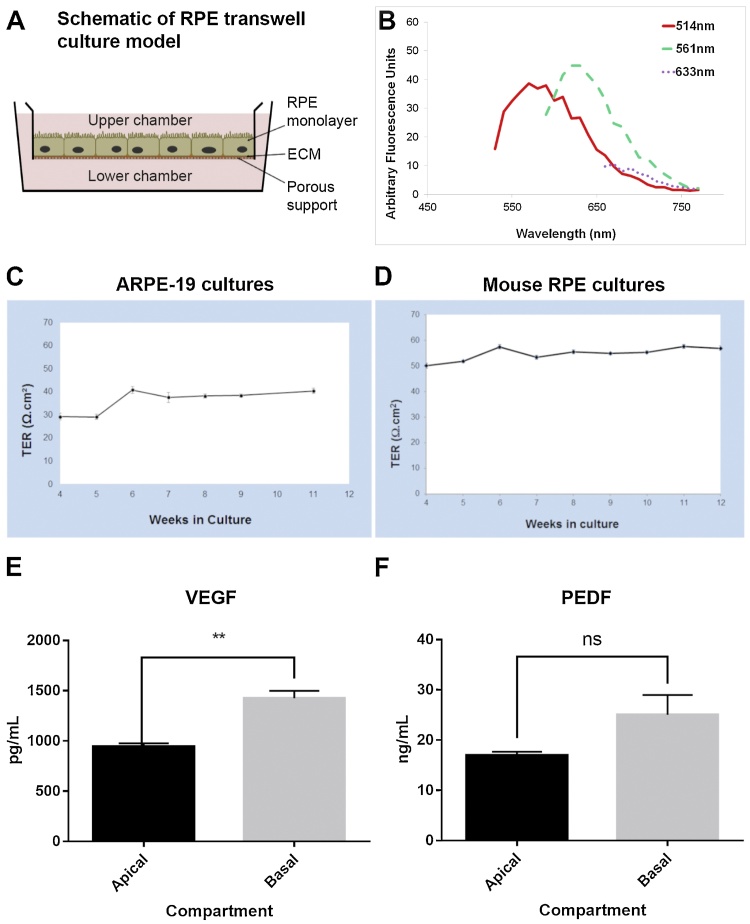
Mature RPE cultures on polyester transwell membranes recapitulate several important features of native RPE. [A] A cross-sectional schematic of the RPE transwell culture. ECM: Extracellular matrix. [B] Primary mouse RPE cells on transwells retained high autofluorescence levels after 20 weeks in culture with notable spectral peaks at 514 nm and 561 nm. [C] Once established, ARPE-19 monolayers on transwells maintained a consistently stable *trans*-epithelial barrier (TER) averaging 36.3 ± 1.1 Ω per cm^2^. Measurements recorded in n = 3 wells in triplicate per week. [D] Primary mouse RPE cells on transwells also showed stable TER values that were comparatively higher, averaging 54.8 ± 0.69 Ω per cm^2^ over a similar period. Measurements recorded in n = 3 wells in triplicate per week. [E] 12 week old ARPE-19 cells on transwell membranes displayed preferential VEGF secretion via the basolateral surface. The ratio of VEGF secretion was 3:1 in favour of the basolateral surface, p = 0.0039 (**). [F] Levels of PEDF secreted via the respective apical and basolateral RPE surfaces showed no statistical significance (ns), although higher quantities were recorded in the basal transwell chamber. Conditioned media collected from n = 3 wells with measurements in triplicate.

*Next, we evaluated the capacity of RPE cells to either retain or develop autofluorescence in transwell cultures (Fig. 5A), depending on whether they are primary RPE or ARPE-19 cells, respectively. Mouse RPE cells were observed to retain relatively high amounts of autofluorescent material even after 20 weeks in culture with notable peaks recorded at 514 nm and 561 nm (Fig. 5B). No autofluorescence signals were detected in ARPE-19 cultures (data not shown). A key property of well-established RPE monolayers is their ability to maintain an effective electrochemical gradient between the retina and the outer retinal vasculature (Marmor and Wolfensberger, 1998, Pfeffer and Philp, 2014, Reichhart and Strauss, 2014). This can be measured in-vitro as a *trans*-epithelial electrical resistance (TER) between upper and lower chambers of a transwell dish separated by a mature and confluent RPE monolayer. We therefore carried out longitudinal TER recordings of ARPE-19 and primary mouse RPE cultured on polyester transwell membranes, which were found to be both robust and stable for approximately 2 months (Fig. 5C–D). The respective TER measurements averaged over this period were 36.3 ± 1.1 Ω per cm2 for ARPE-19 cultures and 54.8 ± 0.69 Ω per cm2 for primary mouse RPE cultures. We next determined whether these RPE cultures were capable of directionally secreting two proteins critical to retinal homeostasis (Fig. 5E–F). Compartmentalisation of conditioned media into apical and basal chambers in transwell inserts cultured with ARPE-19 allowed the quantification of vascular endothelial growth factor (VEGF) and pigment epithelial growth factor (PEDF) as follows. VEGF; [apical]: 941.8 ± 35.35 pg/mL (n = 3), [basal]: 2851.69 ± 145.41 pg/mL (n = 3), p = 0.0039 (**). PEDF; [apical]: 16.95 ± 0.72 ng/mL (n = 3), [basal]: 25.05 ± 3.92 ng/mL (n = 3), p = 0.0656. We also quantified VEGF levels secreted by primary mouse RPE which had been growing on the electrospun scaffolds for ∼9 months. These yielded values of 702.4 ± 27.3 pg/mL (n = 5).

## 4 Discussion

The ability to recreate authentic tissues under culture conditions has advanced steadily due to improved formulations of commercially available culture media, developments in creating better substrates, cell isolation methods as well as generating cells more suitable for such studies. Attempts to faithfully reproduce in-vitro RPE has benefited from all of the above such that RPE from sources as diverse as embryonic/adult-induced pluripotent stem cells (Brandl et al., 2014, Vugler et al., 2008), human (Sonoda et al., 2009, Hu and Bok, 2001), rodent (Fernandez-Godino et al., 2016, Heller et al., 2015) and porcine RPE^15^, as well as cell lines including ARPE-19 (Dunn et al., 1996), RPE-J (Nabi et al., 1993) and D407 (Davis et al., 1995) are now routinely cultured on a variety of substrates such as Matrigel™ and transwell inserts. These efforts have produced surrogate RPE monolayers in culture with varying degrees of success (Pfeffer and Philp, 2014). Throughout life, the RPE monolayer maintains a dynamic and reciprocal relationship with the underlying BrM; of which the latter plays a key role apically in the proliferation, migration, differentiation, and cell-to-cell communication of RPE cells (Booij et al., 2010, Bhutto and Lutty, 2012), but also an equally complex role basally with the underlying choriocapillaris (Booij et al., 2010, Ugarte et al., 2006). Histologically, the BrM may be classified as an acellular pentalaminar structure (Hogan, 1961) consisting of: (1) The RPE basement membrane, which is continuous and is of approximately 0.14–0.15 μM in thickness consisting of collagen IV, laminin, fibronectin, heparin sulphate and chondroitin (Booij et al., 2010). (2) An inner collagenous layer of 1.4 μM thickness in the young (Guymer et al., 1999); organised into a multi-layered grid-like structure of collagens I, III, V and embedded with glycosaminoglycans and complement proteins (Booij et al., 2010). (3) An elastin layer of ∼0.8 μM thickness in the young; organised into several stacked layers. In addition to elastin, this layer also contains collagen VI and fibronectin (Booij et al., 2010). (4) An outer collagenous layer of 0.7 μM thickness in the young, which is largely identical to the inner collagenous layer (Marmor and Wolfensberger, 1998). (5) The basement membrane of the choriocapillaris constitutes the final BrM layer, which has an average thickness of 0.14 mm in the young; largely consists of laminin, heparan sulphate as well as collagen IV, V and VI (Aisenbrey et al., 2006). The choriocapillaris basement membrane is however discontinuous due to endothelial fenestrations or pores of the adjacent choriocapillaris (Altunay, 2007). In summary, the BrM forms a porous scaffold with each layer made up of distinct materials with different biophysical characteristics allowing free passage of solutes and molecules between the RPE and underlying choroid (Booij et al., 2010, Bhutto and Lutty, 2012). If authentic RPE monolayers are to be generated in-vitro, the supporting substrate must possess salient biophysical characteristics of native BrMs. Such in-vitro BrM mimics therefore has to pass 2 major challenges. In the first instance, the substrate has to support the attachment, proliferation and formation of RPE monolayers and thus facilitate the generation of functionally-competent RPE which has a similar structure to native RPE. Secondly and ideally for translational purposes, this substrate should then double-up as a scaffold on to which a sheet of functional RPE could be transplanted into patients.

Cell-replacement therapy has garnered considerable interest (Treharne et al., 2011, Heller and Martin, 2014, Thomson et al., 2011), particularly in the wake of recent developments in stem cell biology (Kaewkhaw et al., 2016). In this first study of its kind, we evaluated the capacity of two different synthetic BrM substrates to reproduce and sustain RPE monolayers in-vitro. Our experiments were designed to address several important questions; (1) what are the key considerations when designing a new BrM substrate on which realistic and functional RPE monolayers can be generated in-vitro? (2) Can such models be used to faithfully study dynamic disease processes? (3) What types of materials make a suitable prosthetic support for RPE cell-transplantation? The possibility of a single material forming the ideal RPE substrate for modelling diseases as well as acting as a prosthetic support for cell-replacement therapy may be low. Rather, a given type of substrate may be more suited for one purpose rather than another. For instance, the simplicity of polyester membranes in transwells or use of biological components in Matrigel™ is likely to exclude these from being considered for transplantation therapy. However, as many investigators have elegantly demonstrated, these substrates still act as powerful tools for modelling RPE in-vitro (Ahmado et al., 2011, Pfeffer and Philp, 2014, Sekiyama et al., 2012). In this manuscript, we addressed these salient questions by evaluating widely-utilized polyester membranes as well as a novel electrospun substrate developed in our laboratory (Thomson et al., 2011, Treharne et al., 2012, Ward et al., 2014, Lotery et al., 2016, Fernandez-Godino et al., 2016, Hussain et al., 2011, Hall and Abrams, 1987, Krohne et al., 2010, Reichhart and Strauss, 2014, Brandl et al., 2014, Vugler et al., 2008).

We first tested the ability of commercially available polyester transwell membranes permeated with 0.4 μM pores to support RPE monolayers for up to several months in culture. Primary mouse RPE and ARPE-19 cultured on these membranes formed a specialised surface by producing apical microvilli that projected into the culture media. These well-defined microvilli were numerous but somewhat shorter at ∼5 μm compared to microvilli expressed in native RPE which are reported to extend for as long as half the outer segment (Bonilha et al., 2006). Such discrepancies may be due to lack of outer segment availability with which native RPE microvilli intimately associate. Shortening of apical microvilli is likely to diminish the total surface area of cultured RPE, thus altering the 3:1 apical to basal proportions reported in native RPE (Marmorstein et al., 1998). We observed the basolateral surface of mouse RPE and ARPE-19 cultures to have numerous micro-infolds, which on occasion appeared to form projections into nearby pores in the underlying membrane. RPE cells isolated from human foetal eyes were also reported to project basal processes into Millicell™ pores in a similar manner (Hu and Bok, 2014). Native RPE are also known to form similar projections via their basolateral membranes (Marmor and Wolfensberger, 1998), a process which may be integral to BrM remodelling activities in aged retinas (Marmor and Wolfensberger, 1998, Booij et al., 2010, Bhutto and Lutty, 2012). In addition to allowing free communication with the basal transwell chamber, these pores were also sites at which drusen constituents: vitronectin, clusterin, serum amyloid P component, ApoE and complement (Crabb et al., 2002), accumulated when human RPE were cultured on transwell membranes (Johnson et al., 2011). In another study, cultured ARPE-19 was shown to secrete BrM components, which accumulated uniformly between their basolateral surface and the transwell membrane (Sekiyama et al., 2012). We also observed such sub-RPE deposits, although these were more focal in appearance, and predominantly confined to spaces within RPE basolateral micro-infolds in our cultures. Collectively, these findings suggest that porous polyester transwell membranes enable RPE to form authentic monolayers in-vitro with essential apical and basolateral specialisations including the ability to lay down superficial sub-RPE matrix as well as drusen components.

We were intrigued by how well primary mouse RPE cells could attach, spread and form specialised monolayers on our novel electrospun BrM mimic. In earlier work we had described how a first-generation electrospun scaffold composed of methyl methacrylate and poly(ethylene glycol) methacrylate was synthesised and modified to facilitate cell attachment. This proved suitable for culturing ARPE-19 cells for up to 2 weeks (Treharne et al., 2011, Thomson et al., 2011, Treharne et al., 2012). In developments since then, we substantially refined its properties, such that the second-generation electrospun scaffold used in the current experiments were different in several key aspects. The average fibre diameter was reduced from 1.9 μm to 0.1-0.8 μm, whilst the overall thickness of the electrospun scaffold was condensed from ∼50 μm to12.3 μm (Ward et al., 2014). The new scaffold is therefore somewhat closer to the reported thickness of human BrM, which varies between 2 and 4.7 μm, depending on its relative position in the posterior eye as well as age of the donor (Booij et al., 2010, Bhutto and Lutty, 2012, Ramrattan et al., 1994). These changes are reflected in the diffusion capacities of the improved scaffold, which was similar to human BrM preparations. The chemical composition of our scaffold between successive developments remains unchanged. However, we increased the Young’s modulus of the second generation scaffold such that it was much closer to the human BrM. We also dispensed with RGD peptides (Ward et al., 2014), since primary mouse RPE cells were found to readily attach and spread on the new scaffold surface devoid of these cell-attachment modifications (Lotery et al., 2016). During the first few weeks after RPE seeding, we observed occasional gaps in the monolayer, which we speculate to be caused by lack of suitable attachment-fibres in the vicinity. However, in cultures older than 4 months these spaces had largely disappeared, presumably as extracellular material synthesised by adjacent cells created a suitable substrate with nearby fibres to support RPE within such gaps. Our electrospun scaffold also enabled mouse RPE to form specialised apical membranes with abundant microvilli that projected into the culture media. Of particular interest was how these RPE appear to have secreted an extracellular matrix on the fibre meshwork which has a superficial likeness to an acellular BrM. These images were captured using a freeze-drying SEM protocol to visualise the RPE-BrM assembly from underneath the electrospun scaffold. We also observed what appeared to be focal deposits of extracellular material in amongst the fibre spaces, resembling the accumulation of sub-RPE drusen in BrMs of aged and AMD donor eyes (Curcio and Millican, 1999, Rudolf et al., 2008). Scaffolds incubated in only conditioned media as controls showed no evidence of any extracellular material. The appearance of RPE-synthesised extracellular material within the scaffold was noteworthy as it may enable in-vitro generation of sub-RPE deposits such as basal linear deposits between the RPE and the inner collagenous layer of BrM, or indeed an opportunity to study the biogenesis of drusen that is associated with macular pathology (Booij et al., 2010, Curcio and Millican, 1999). Our future experiments will attempt to identify these apparent sub-RPE deposits, and study how they evolve over time. As drusen components are considered to originate from either the RPE or the choroidal vasculature or most likely a combination of both (Crabb et al., 2002), such studies may also help identify drusen constituents produced by RPE cells alone.

We then assessed features of long-term RPE cultures on their respective substrates. Mature ARPE-19 cultures on polyester transwell membranes expressed ZO-1 junctional complexes as well as the RPE specific protein RPE65. We also observed apically localised NA^+^/K^+^ATPase which is associated with photo-transduction (Ames et al., 1992). It is reported that this transporter will not become apically localised unless ARPE-19 cultures were pigmented and likely to be more differentiated (Ahmado et al., 2011). Indeed, ARPE-19 and mouse RPE on polyester membranes as well as mouse RPE on electrospun scaffolds showed signs of pigmentation, another indicator of highly differentiated RPE (Boulton, 2014). We next studied the features of primary mouse RPE on our electrospun scaffold. These RPE also adopted a characteristic hexagonal morphology and expressed ZO-1, NA^+^/K^+^ATPase, as well as RPE65. We studied how these cells interacted with fibres in the electrospun scaffold. Points of RPE cytoskeleton in contact with the underlying fibres could be identified via expression of FAK; a non-receptor tyrosine kinase which links integrin or growth factor signals to the intracellular cytoskeleton (Parsons, 2003). It appears that mouse RPE attach to electrospun fibres using the same mechanisms that other primary RPE cells employ in forming attachments to underlying substrates (Opas, 1985). We next determined the functional capabilities of RPE cells on their respective substrates. The ability to bind, phagocytose and proteolytically degrade POS on a daily basis is a unique feature of RPE cells (Marmor and Wolfensberger, 1998, Mazzoni et al., 2014). This is an area which has garnered considerable attention as impairment of these processes is associated with age and diseases such as AMD and RP (Kaarniranta et al., 2013, Sun et al., 2007). Several elegant reviews have described the molecular machinery associated with this process, including in ARPE-19 cells cultured on transwell membranes (Mazzoni et al., 2014, Ferrington et al., 2016). For the first time we show that primary mouse RPE cells on electrospun scaffold are capable of binding POS via MerTK and αVβ5 receptor-mediated mechanisms. The time course of POS internalisation, as well as proteolytic processing, which occurred between 6 and 8 h, was in-line with fast kinetics reported in primary rat RPE cells (Mao and Finnemann, 2013). Although we did not directly assess proteolytic degradation per se, reduced numbers of FITC-POS fluorescent puncta as well as diminished signals from internalised MerTK and αVβ5 receptors after 3 h indirectly suggested degradation of internalised cargos. These processes are known to occur within RPE lysosomes-phagosomes during terminal stages of POS internalisation (Mazzoni et al., 2014, Ferrington et al., 2016). Our results also demonstrated that the electrospun scaffold can support RPE that is capable of efficiently carrying out a key function which is directly influenced by the age of native human BrMs (Sun et al., 2007, Moreira et al., 2015). Parallel experiments in ARPE-19 grown on transwells showed polyester membranes to support RPE monolayers capable of POS binding/internalisation. Timelines for these activities were also consistent with phagocytosis carried out by this cell-line (Mazzoni et al., 2014, Mao and Finnemann, 2013). We next assessed how, following receptor-mediated phagocytosis, RPE cells might internalise ingested POS; a process considered to occur via the endocytic/lysosomal pathway. We report for the first time that ingested POS co-localised with Rab-5 early endosomes as well as Rab-7 late endosomes before being trafficked to late-stage endocytic compartments. Although the terminal stages of POS clearance in LAMP-positive lysosomes and LC3-positive autophagy vesicles have been well-documented before (Kaarniranta et al., 2013, Ferrington et al., 2016), our studies suggest that at least a proportion of internalised POS is initially trafficked via Rab-5 and Rab-7endosomes. The pulse-chase feeding assay (Hall and Abrams, 1987) increased the likelihood that most POS cargos were phagocytosed within a narrow window. Once internalised, the timeframes in which POS was trafficked via the respective compartments also conformed to reported timelines in which POS cargoes were processed by RPE (Mazzoni et al., 2014). We also measured the relative sizes of endocytic compartments which showed a gradual increase in vesicle dimeter as early endosomes matured to form late endosomes, lysosomes and autophagic bodies. These measurements were consistent with sizes of endosomes, reported to be between 200 and 500 nm (Roberts et al., 1999), which expand to 500nm–1 μm in diameter as mature lysosomes (Huotari and Helenius, 2011). We observed the appearance of POS-positive LC3b autophagy vesicles, approximately 48 h after POS challenge in instances where the conjugated FITC tag appeared to have survived late-stage proteolytic digestion. Elevated levels of autophagic vesicles are reported in aged RPE of mice and human donor eyes without AMD (Ferrington et al., 2016). However with onset of disease, the proteolytic capacity of RPE becomes overwhelmed resulting in a decline/impairment in autophagy, an observation supported by diminished numbers of autophagic vesicles in AMD donor RPE compared to heathy age-matched controls (Mitter et al., 2014). Our results suggest that RPE cultured on suitable substrates can be used to model such subtle disease-related pathology.

High autofluorescence is a characteristic feature of native RPE (Boulton, 2014). Autofluorescence increases with age as partially digested POS, damaged organelles and proteins referred to as lipofuscins accumulate within RPE phagosome-lysosomes and autophagosomes (Ferrington et al., 2016). Such lipofuscin-filled vesicles occupy approximately 19% of the total cytoplasmic volume by the eighth decade of life (Boulton, 2014, Feeney-Burns et al., 1984). These are some of the features which some in-vitro RPE cultures have found difficult to reproduce. We report here for the first time that our long-term cultures of primary mouse RPE retain high levels of autofluorescence even without addition of POS. The presence of autofluorescence peaks at 514 nm and 561 nm are noteworthy, as they correspond to spectral emissions characteristic of lipofuscin and A2E reported to be between 450 and 590 nm (Biesemeier et al., 2011) and 565–570 nm (Sparrow et al., 1999), respectively. However, it should be noted that others have indicated lipofuscin to have a much broader spectral range with peaks at 560 nm, 600 nm and 625 nm, distinct to spectral peaks in POS fed-RPE cultures at 425 nm and 525 nm (Boulton, 2014, Boulton et al., 1989). This suggests differences in de-novo generated autofluorescent material in cultured RPE. At this stage, we are unable to speculate on the precise aetiology/identity of 514 nm, 561 nm and 633 nm autofluorescence peaks in our primary mouse RPE cells, other than they may have been retained when cells were isolated from native tissue and/or formed by intracellular activities after 20 weeks in culture. It is worth noting that autofluorescence was reported to increase in long-term bovine and human primary RPE cultures without the addition of POS substrate (Burke and Skumatz, 1998, Wassell et al., 1998); an increase thought to be due to basal autophagy activities which can make significant contributions to lipofuscin accumulation in cultured RPE (Mitter et al., 2012). Future experiments will evaluate the extent of basal autophagy in our long-term RPE cultures. We also compared the ability of primary mouse RPE and ARPE-19 to form a stable electrochemical gradient on polyester transwell membranes. The ability to maintain an effective electrochemical separation between the retinal environment and the outer vasculature is a key feature of the RPE(Marmor and Wolfensberger, 1998, Lehmann et al., 2014). Numerous studies have demonstrated that ARPE-19 cells cultured on transwell membranes exhibit diminished electrochemical gradients (Luo et al., 2006). The TER values of our long-term ARPE-19 cultures averaged 36.3 ± 1.1 Ω per cm^2^, in-line with values reported by others for this cell line (Pfeffer and Philp, 2014, Kannan et al., 2006, Ahmado et al., 2011, Luo et al., 2006, Geisen et al., 2006). Some studies however indicate that ARPE-19 are unable to maintain stable barriers over longer time periods (Geisen et al., 2006), although we found no evidence of this in our cultures. By contrast, primary mouse RPE on transwell membranes were reported to develop TER values of 33 Ω per cm^2^ for up to 15 days, whereupon TER recordings diminished considerably (Geisen et al., 2006). Here we show for the first time that mouse RPE monolayers on similar membranes were capable of stably maintaining average TER values of 54.8 ± 0.69 Ω per cm^2^ over a 2 month period. Next, we assessed the capacity of long-term ARPE-19 on transwells to directionally secrete proteins that are critical to the survival of the choroid as well as the neuroretina. The RPE has been shown to be a major site of VEGF synthesis (Saint-Geniez et al., 2006); a pro-angiogenic protein critical to choroidal perfusion and survival of the choriocapillaris (Saint-Geniez et al., 2009). PEDF is also synthesised by the RPE, but has anti-angiogenic as well as neuroprotective properties (Tombran-Tink et al., 1995). We measured the respective quantities of VEGF and PEDF proteins secreted by apical and basolateral surfaces of ARPE-19 cells. Although primary RPE cells have been shown to preferentially secrete VEGF via their basolateral surface (Blaauwgeers et al., 1999), it is a feature reportedly lost in some ARPE-19 cultures (Kannan et al., 2006). We specifically measured VEGF-A, which has been implicated in the vascular form of AMD (Marneros, 2013). We show that under our culture conditions, mature ARPE-19 monolayers preferentially secreted VEGF via its basolateral surface. The combined VEGF levels from both apical and basal chambers, which measured approximately 3700 pg/mL in a 3:1 (basal to apical ratio), were within VEGF levels secreted by primary RPE cells isolated from human donors (Blaauwgeers et al., 1999). However, the availability of VEGF quantities over a given time period must be considered with caution as the biological half-life of VEGF-A 165 is reported to be only 90 min (Kleinheinz et al., 2010). There are reports that ARPE-19 cells do not express PEDF (Klimanskaya et al., 2004). However, our results show that mature ARPE-19 cultures secrete abundant quantities of PEDF via both apical (16.95 ± 0.72 ng/mL) and basolateral surfaces (25.05 ± 3.92 ng/mL). These figures were lower than PEDF quantities secreted by primary monkey RPE (Becerra et al., 2004), but higher than primary human RPE cells (Falk et al., 2012). These discrepancies could be due to differences between primary RPE vs. RPE cell lines, different substrates, contrasting quantification methods as well as changes to RPE which may occur under in-vitro conditions. Quantification of VEGF secreted by ∼9 month old mouse RPE cultured on electrospun scaffolds revealed this substrate to support long-term monolayers of primary RPE that were capable of synthesising/secreting this important protein. Future studies will incorporate mouse RPE-BrM assemblies on electrospun scaffolds into a transwell-like system so that apical vs. basolateral secretion profiles can be separately quantified. This will also enable the quantification of TER values, thus help evaluate how effectively the alternative electrospun substrate can support the establishment of effective RPE monolayers in-vitro.

## 5 Conclusions

In this first report, we evaluated the capacity of two very different BrM substrates to support the in-vitro development of RPE monolayers. Of the two substrates, one is a widely utilized polyester transwell membrane, whilst the other is a novel electrospun scaffold generated in our laboratory. Our findings helped answer some of the questions which we set out to address: (1) what are the key considerations when designing a new substrate on which authentic RPE monolayers can be generated in-vitro? Our data suggest that as long as the potential substrate facilitates the ready attachment, proliferation and migration of adherent cells, authentic RPE cultures may be generated on a substrate with suitably sized pores or gaps in the membrane. We reason that the presence of such gaps, whether in the form of pores within polyester transwell membranes or spaces within the electrospun scaffold is critical for RPE cells, as they appear to recapitulate the porosity of BrM, and thus promote the development of a polarised monolayer with distinct apical and basolateral adaptations. (2) Can ex-vivo RPE models be used to study dynamic disease processes that are difficult to undertake in-vivo? Numerous groups have exploited transwell RPE cultures to study disease processes in-vitro. Our findings provide further evidence of the versatility of the transwell model. We show for instance, how it can be used to produce authentic RPE tissues in a dish that provide mechanistic insights into POS trafficking which becomes impaired in diseases such as AMD and RP. We also show these models may be suitable to study important intracellular activities such as autophagy which contribute to cellular autofluorescence. We also found that our novel electrospun scaffold supports long-term cultures of bona-fide RPE monolayers under culture conditions. Moreover, these scaffolds may allow for more realistic and complex studies of RPE-BrM interactions such as the generation of drusen-like sub-RPE deposits under in-vitro conditions. Electrospinning techniques have also been used by others to generate RPE scaffolds (Xiang et al., 2014, Popelka et al., 2015). However, they do not discuss their potential to study disease-linked features, although their capacity as a cell-delivery system is amply demonstrated. (3) What type of materials makes a suitable prosthetic support on which a sheet of functional RPE could realistically be transplanted? As a copolymer solely generated from chemical compounds that are certified to be safe for clinical use by the US Food and Drug Administration (Treharne et al., 2012, Ward et al., 2014), our novel electrospun scaffold supports authentic, primary RPE monolayers for up to 1 year in culture. Their robustness/longevity and ease of manipulation makes these amenable to transport and shipping. Their reproducibility and ability to scale-up this process makes these electrospun RPE-BrM assemblies hold great promise for future therapeutic applications. Findings from this model in particular have prompted further questions. For instance, do apparent sub-RPE focal deposits within the electrospun fibre meshwork represent genuine drusen-like deposits? What is the TER value of RPE monolayers on electrospun scaffolds? To what extent can these RPE cells secrete important proteins in a polarised manner? Future experiments in our laboratory will address these questions. Our findings presented here however, demonstrate how authentic RPE tissues can be recapitulated on two different substrates, and successfully maintained over long periods in culture.
