# Supplementary material for: Ex-vivo models of the Retinal Pigment Epithelium (RPE) in long-term culture faithfully recapitulate key structural and physiological features of native RPE
Source: Tissue Cell. 2017 Aug;49(4):447–60. doi: 10.1016/j.tice.2017.06.003 (PMC5545183; doi:10.1016/j.tice.2017.06.003)
Supplement: Supplementary file 1 [file mmc1.pdf]

Supplementary information

Sample number	Voltage (kV)	Flowrate (mL/Hr)	Distance to collector (cm)	Average fibre thickness (μm)	Presence of polymeric beads	Signs of scaffold breakage
1	15	0.05	15	0.5-2	Yes	Yes
2	15	0.1	15	1.5-3	Yes	No
3	15	0.2	15	1.5-3	Yes	No
4	15	0.5	15	1.5-3	Yes	No
5	15	1	15	3-5	Yes	Yes

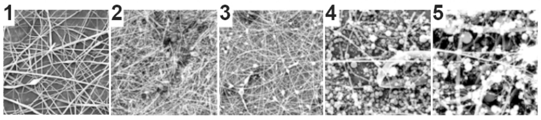

Table S1: Effects of increased flowrates on electrospinning of the second generation scaffold. Notice the formation of undesirable polymeric beads amongst fibres at flowrates higher than 0.05mL/Hr.

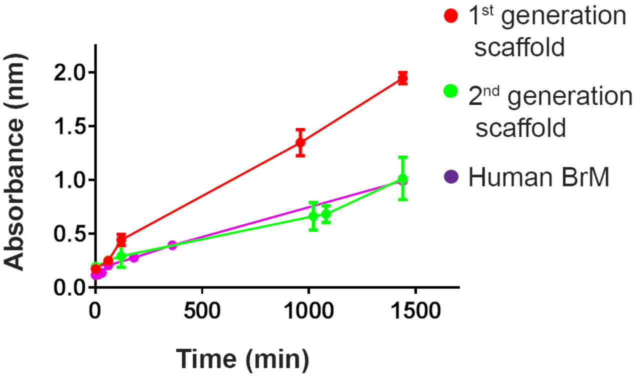

Fig. S1: Representative diffusion graph. The membrane was inserted into an Ussings chamber where one compartment contained a solution of 40 kDa FITC-Dextran. The ability to allow free passage of fluorescent particles into the adjacent reservoir through respective membranes were quantified by measuring the absorbance using a spectrophotometer. Notice the similarities between the second generation electrospun scaffold and human BrM. N=3 for all experiments.

	Sample number	Voltage (kV)	Flowrate (mL/Hr)	Distance to collector (cm)	Average fibre thickness (μm)	SEM +/-	Polymeric beads	Scaffold breakage
	1	15	0.05	15	1.14	0.45	Yes	Yes
	2	15	0.1	15	1.64	0.41	Yes	No
	3	15	0.2	15	1.7	0.40	Yes	No
	4	15	0.5	15	1.99	0.40	Yes	No
	5	15	1	15	3.01	0.67	Yes	Yes
	6	15	0.05	15	0.77	0.35	Yes	No
	7	20	0.05	15	0.79	0.33	Yes	No
	8	25	0.05	15	0.55	0.29	No	No
	9	30	0.05	15	0.56	0.24	No	Yes
*	10	35	0.05	15	0.42	0.13	No	No
	11	15	0.1	15	1.91	0.26	No	Yes
	12	20	0.1	15	1.74	0.31	Yes	No
	13	25	0.1	15	1.11	0.22	Yes	No
	14	30	0.1	15	1.46	0.19	Yes	No
	15	35	0.1	15	0.98	0.32	Yes	No
	16	15	0.2	15	2.24	0.66	Yes	No
	17	20	0.2	15	2.01	0.58	Yes	No
	18	25	0.2	15	1.55	0.68	Yes	No
	19	30	0.2	15	0.99	0.44	Yes	No
	20	35	0.2	15	0.74	0.32	Yes	No
	21	15	0.5	15	2.27	0.32	Yes	No
	22	20	0.5	15	1.94	0.72	Yes	No
	23	25	0.5	15	1.34	0.41	Yes	No
	24	30	0.5	15	1.15	0.36	Yes	No
	25	35	0.5	15	0.71	0.22	Yes	No

Table S2: The combined effects of increased voltage and flowrates on electrospinning of the the second generation scaffold.Optimal parameters are highlighted in purple (*). N =5 for each data point.

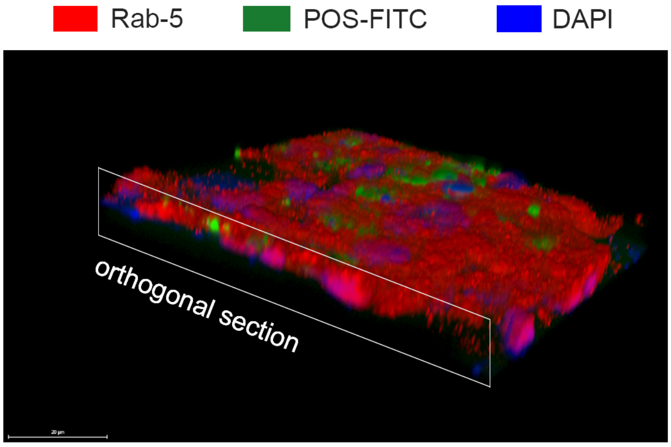

Fig. S2: Generation of orthogonal sections from confocal z-stacks. Orthogonal views through RPE monolayers is an effective means to show dynamic molecular events such as receptor-mediated POS binding and intracellular trafficking of cargos.

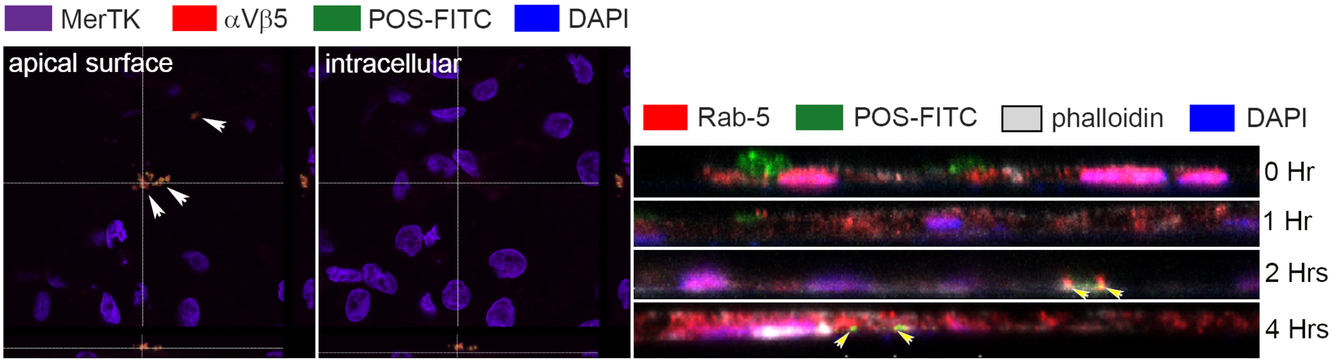

Fig. S3: POS internalisation and trafficking by ARPE-19 on polyester transwell membranes. Lefthand panels show on-face and orthogonal views of cargo (white arrows) binding to receptors on ARPE-19. These cargo-receptor complexes exclusively co-localise to the RPE surface and are absent in deeper layers of the cell. Righthand panel shows orthogonal views of POS internalisation into Rab-5 positive vesicles over time. POS within Rab-5 compartments are visible as yellow puncta (arrows).
